# Comparison of residual silicone oil index after removal of silicone oil with fluid-air versus oil-fluid exchange

**DOI:** 10.12669/pjms.39.2.6243

**Published:** 2023

**Authors:** Amna Rizwan, Rana Muhammad Mohsin Javaid, Sidrah Latif, Muhammad Suhail Sarwar, Asad Aslam Khan

**Affiliations:** 1 Amna Rizwan, Eye Unit-III, Mayo Hospital Lahore, Pakistan; 2 Rana Muhammad Mohsin Javaid, Eye Unit-III, Mayo Hospital Lahore, Pakistan; 3 Sidrah Latif, Eye Unit-III, Mayo Hospital Lahore, Pakistan; 4 Muhammad Suhail Sarwar, Eye Unit-III, Mayo Hospital Lahore, Pakistan; 5 Asad Aslam Khan, Eye Unit-III, Mayo Hospital Lahore, Pakistan

**Keywords:** Silicone oil, B-scan, Fluid-air exchange, Oil-fluid exchange, Silicone oil index, Imagej software

## Abstract

**Objectives:**

To compare the effectiveness of fluid-air exchange with silicone oil-fluid exchange in reducing the residual silicone oil (SO) droplets after the removal of SO.

**Methods:**

This was a prospective, quasi-experimental study conducted from October 2021 to February 2022 at Eye Unit-III, COAVS, Mayo Hospital, Lahore. Sixty-one patients with siliconized eyes underwent removal of SO with two different techniques and were divided into fluid-air exchange and oil-fluid exchange groups. To quantify the residual silicone droplets objectively, B-scan echographic images were analyzed within seven days of surgery. Silicone oil index (SOI) which is the amount of residual SO droplets/vitreal area in the images was calculated with the help of imagej software.

**Results:**

The residual SOI of the fluid-air exchange group (0.99 ± 1.76%) was significantly lower than the oil-fluid exchange group (3.25 ± 3.85%). The SOI is positively correlated with the duration of tamponade, preoperative intraocular- pressure and axial length. Persistent IOP elevation post-operatively was seen in 16.67% individuals in the fluid-air exchange group and 54.8% individuals in the oil-fluid exchange group.

**Conclusion:**

Fluid-air exchange group was found to be superior in reducing residual SO droplets than the oil-fluid exchange group.

## INTRODUCTION

Silicone oil (SO) was first introduced as an internal tamponade in vitreoretinal surgery in the 1960s.^1^ It has since then developed into a valued method used in retinal detachment (RD) surgery. SO is used in complex rhegmatogenous retinal detachment (RRD) especially with severe proliferative vitreoretinopathy (PVR), giant retinal tears, proliferative diabetic retinopathy (PDR), traumatic RD, endophthalmitis, macular hole surgery, RD associated with choroidal coloboma and complicated pediatric RDs.^2^-^4^ Complications of SO include glaucoma, chronic hypotony, cataract formation, recurrent RD, SO emulsification, keratopathy and migration of SO in the anterior chamber.^5^

In practice, it is difficult to make the judgment call to remove SO. As a reference, in the Silicone study, SO was removed after a minimum of eight weeks. In general, however, SO is typically removed within six months following surgery.^6^ Various active or passive methods may be employed for removing SO, with active ones generally preferred.^7^,^8^ Complete removal of silicone oil (ROSO) is seldom possible. Emulsified droplets adhere to the ciliary recess, zonules and posterior aspects of the iris.

Many techniques are being used to remove emulsified silicone oil droplets. The suction method used can involve active aspiration or passive perfusion.^9^ One method is by silicone oil-fluid exchange (OFX).^9^ Other active method is based on repeated fluid-air exchange (FAX) cycles. In this method, the air replaces the SO in the vitreous cavity.^9^ In practice, however, several SO droplets are generally seen post-procedure.

The relative efficacy of these methods is traditionally quantified either in terms of surgical time or via post-operative slit-lamp examination which is a rather crude indicator of the removal. B-scan ultrasonography, on the other hand, provides us with a reliable quantitative tool to measure the efficacy of various methods. B-scan ultrasonography employs Rayleigh scattering to exaggerate the SO residue and, thus, enables us to make a good estimate of the residual SO droplets.^10^ This study investigated the efficacy of FAX technique vs OFX technique in reducing residual SO droplets using B-scan ultrasonography.

## METHODS

A quasi-experimental study was conducted at Eye Unit-III, COAVS, Mayo Hospital, Lahore from October 2021 to February 2022 after approval from the ethical review board (No.COAVS/1106/2021, Date: 12-10-2021). A total of 61 patients were included by non-probability convenient sampling by using the level of significance as 95% and power of test as 80%.^10^ All patients who underwent surgery for ROSO were included except patients with corneal opacities, SO tamponade of more than four years, emulsification of silicone oil, any event of post-operative vitreous hemorrhage, or low-quality ultrasonographic images. A written informed consent with demographic information was collected from each patient. Included eyes underwent slit-lamp examination of the anterior segment, intraocular pressure (IOP) measurements with a Goldmann applanation tonometer, fundus evaluation, and pre-operative axial length (AL) measurements. History, diagnosis of the disease, age, gender, the status of lens and duration of the SO tamponade were noted.

### Surgical Technique:

All surgeries were done by the same surgeon. Three port sclerotomy with 23-gauge trocars were made with infusion port placed inferotemporally and two superior ports for aspiration. B.E.S (Balanced Electrolyte Solution) was allowed to replace globe volume as SO was aspirated with a bottle height of 80 centimeters above the eye. The bulk of the SO was removed from the sclerotomy site with a 10 ml syringe by pulling the plunger of the syringe to the end to create maximum negative pressure.

**Fig.1:**
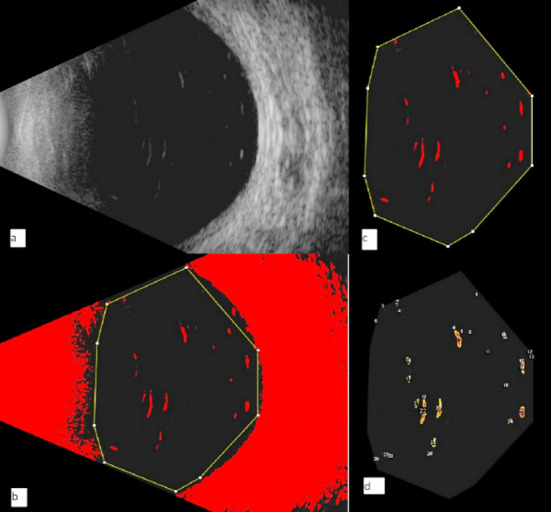
Representation of image processing using imageJ software to quantify residual SO droplets. a. Ultrasound B-scan image of a patient. b. Binarization of the image to highlight signals from the residual SO droplets in “color threshold” mode. c. vitreous cavity area was demarcated. d. Image showing number and area of residual SO droplets.

After the bulk removal, patients were divided into two groups; a FAX group in which two to three fluid-air exchange cycles were done, and an OFX group in which the posterior segment was washed continually with infusion fluid for at least three minutes. After both techniques, the fundus was examined. The anterior chamber was washed in both techniques if required. Sutures were placed to close the sclerotomies. B-scan ultrasonography was performed (by the same ophthalmologist) with a standard ultrasonographic device within seven days of surgery or when the air was absorbed. To remove observer bias, a total of three B-scan images were taken and the mean was used. Patients were further asked to look either towards right or left shoulder to avoid the lenticular and intraocular lens shadows. B-scan machine used was compact touch, with 90 gain, zoom 170 and time-gain compensation (TGC) zero. To quantify the residual SO droplets objectively, a binarization method was applied to the B-scan images using color threshold adjustment as shown in Fig.1. These images were assessed using ImageJ software (ImageJ version 1.47, National Institutes of Health, Bethesda, MD; available at: http://imagej.nih.gov/ij/). The ratio of the sum of the SO droplet areas to that of the vitreous cavity is defined as silicone oil index (SOI) and was calculated using the formula.^10^

### Statistical analysis:

SPSS 26 was used to analyze the data. Mean and standard deviation were calculated for quantitative data while percentages and frequencies were calculated for qualitative data. The relationships between SOI and the different ocular parameters were determined by Pearson and spearman’s correlation tests. The SOIs between the OFX group and the FAX group were compared using the Mann–Whitney U test. A *p*-value ≤0.05 was considered significant.

## RESULTS

A total of 61 individuals including 45 males (73.8%) and 16 females (26.2%) were included, with a mean age of 44.72 ± 17.06 years. Out of the 61 eyes, 45 (73.8%) had been treated for an RRD, 13 eyes (21.3%) for a PDR and three (4.9%) for endophthalmitis. Twenty-four individuals (39.3%) were diabetic, and 21 (34.4%) were hypertensive. The mean AL was 24.27 ± 2.22 mm, and 16 eyes were phakic, 40 were pseudo-phakic and five were aphakic. Demographic findings of FAX and OFX groups were shown in Table-I.

**Table-I:** Fluid-air exchange and oil-fluid exchange group parameters.

Variables	Fluid-Air exchange (n = 30)				Oil-Fluid exchange (n = 31)			P-value
Age	44.50 ± 15.06				44.94 ± 19.04			0.608^a^
Gender (M/F)	21/9				24/7			0.510^b^
Preoperative IOP	24.00 ± 7.10				28.39 ± 9.83			0.109^a^
AL	23.61 ± 1.72				24.92 ± 2.49			0.036^a^
SOI	0.99 ± 1.76				3.25 ± 3.85			0.004^a^
SOI Correlations	Preoperative IOP	Pearson’s r= 0.709		P<0.001	Preoperative IOP	Pearson’s r= 0.533		P=0.002
	Duration of tamponade	Spearman’s r = 0.496		P=0.005	Duration of tamponade	Spearman’s r = 0.385		P=0.033
	Axial length	Pearson’s r = 0.265		P=0.157	Axial length	Pearson’s r = 0.394		P=0.028
	Postoperative IOP	Pearson’s r = 0.787		P<0.001	Postoperative IOP	Pearson’s r = 0.533		P=0.002
Visual Impairment	Preoperative		Postoperative		Preoperative		Postoperative	
Mild Moderate Blind	0		1		1		2	
	11		18		5		14	
	19		11		25		15	

a = Mann-Whitney U test;

b = chi-square test; IOP - intraocular pressure; AL - axial length; SOI - silicone oil index.

Using the WHO criteria, preoperatively, one (1.6%) individual had mild visual impairment (VA≤6/18), 16 (26.2%) had moderate impairment (VA > 6/18 to 6/60), and 44 (72.1%) individuals were blind (VA< 3/60), as shown also in Table 1. The mean IOP was 26.23 ± 8.804 mmHg. SO tamponade duration was less than six months in nine individuals, up to one year in nine, and more than one year in 43 individuals.

Postoperatively, three (4.9%) individuals had mild impairment, 32 (52.5%) had a moderate impairment, and 26 (42.6%) were blind. The mean IOP was 22.05 ± 9.19 mm. Across the sample, the mean of SOI was 2.14 ± 3%. For the FAX group, the mean was 0.99 ± 1.76%. For the OFX group, the mean was 3.25 ± 3.85%. The Mann Whitney-U test was used to compare means of SOI in FAX and OFX groups and was found to be significant (mean rank 24.33 vs 37.45, respectively, p = 0.004).

Pearson and spearman’s correlation was used for the SOI with various ocular parameters where appropriate, as shown in Table-I. It was moderately positively correlated with preoperative IOP (Pearson’s r = 0.600), AL (Pearson’s r = 0.425) and duration of SO tamponade (Spearman’s r = 0.441, p<0.001). Neither gender nor age was associated with SOI. For the AFX group, SOI was strongly correlated with preoperative IOP (Pearson’s r = 0.709) and moderately correlated with duration of SO tamponade (Spearman’s r = 0.496, p = 0.005). It was not related to AL but was strongly correlated with postoperative IOP (Pearson’s r = 0.787).

For the OFX group. SOI was moderately correlated with preoperative IOP (Pearson’s r = 0.533, AL (Pearson’s r = 0.394) and duration of tamponade (Spearman’s r = 0.385, p = 0.033). It was also related to post operative IOP (Pearson’s r = 0.533). Finally, multivariate analysis was used for those variables found to be significant in univariate analysis, as shown in Table-II.

**Table-II:** Multiple linear regression.

Variable		Number of individuals	Univariate analysis			Multivariate analysis		

			Standardized Beta	P-value	95% CI	Standardized Beta	P-value	95% CI
1	***Duration***							
	< 6 months*	9	Reference					
	≥ 6 months	52	0.259	0.044	0.062 - 4.557	0.085	0.410	-1.067 - 2.577
2	Axial length	61	0.425	0.001	0.272 - 0.948	0.241	0.026	0.044 - 0.647
3	***Group***							
	FAX*	30	Reference					
	OFX	31	0.356	0.005	0.713 - 3.798	0.161	0.129	-0.304 - 2.339
4	Preoperative IOP	61	0.600	<0.001	0.142 - 0.293	0.476	<0.001	0.096 - 0.249

* =For group, FAX was the reference variable. FAX= fluid-air exchange, OFX=oil-fluid exchange, IOP=intraocular pressure

In the FAX group, improvement in vision was noted in 14 (46.67%) individuals and 16 (53.3%) individuals showed no change in vision. In OFX, vision improvement was noted in 14 (45.2%) individuals, no change in 16 (51.6%) and one (3.2%) showing decreased vision.

Preoperative IOP was between 10-19 mmHg in 13 (21.3%) individuals, 20 - 29 in 29 (47.5%), and ≥30 in 19 (31.1%) people. Post-op IOP was between 10 - 19 mmHg in 35 (57.3%) individuals, 20 - 29 in 11 (18%), and ≥ 30 in 15 (24.5%) people.

The mean IOP difference in the FAX group was -4.266 ± 2.362 mmHg, and for the OFX group, it was -4.09 ± 5. 461. Although they were not found to be significantly different. Persistent IOP elevation defined as at least 22 mm Hg post-operative was seen in five (16.67%) individuals in the FAX group and 17 (54.8%) individuals in the OFX group. (Spearman’s r = 0.397, p = 0.002).

## DISCUSSION

The principal result of our study is that that FAX technique is superior to OFX technique in reducing SO droplets as SOI (0.99 ± 1.76%) of FAX was less than OFX group (3.2 5 ± 3.85%). We have come to this conclusion after a careful analysis of residual silicone oil using B scan ultrasonography aided by ImajeJ software.

In complicated RD surgery, the choice of using an SO tamponade has clear advantages which include a shorter recovery time and quicker visual rehabilitation, no restriction on air travel, and allowance of comfortable post-operative posture. The only drawback is the necessity of a follow-up procedure to remove the silicone oil.^11^ It is important to use the best available method to remove SO as incomplete removal of these small oil droplets can cause complications like secondary glaucoma, keratopathy, cataract, trabeculitis and chronic elevated IOP.^12^

Our principal result of the superiority of SO removal using FAX is consistent with the findings of Yu J et al in which another method (Coulter counter) to measure the number of droplets directly was used.^13^ Also consistent with our results, the superiority of FAX has been variously argued in other studies as well.^9^,^14^ In supine position, when air is injected, SO collects in the macula and forms a thin layer between the infusion fluid and air. A backflush cannula inserted at the level of this oil infusion fluid interface can easily extract all the SO. Another location for small SO residual particles is the retroiridial plane. Flow of air can dislodge these and make the removal possible via the backflush cannula. This mechanism, of course, is not available while using OFX.

However, our findings are contrary to the results of Shiihara et al who concluded that the number of residual SO droplets increase after the FAX process.^10^ Shihaara et al mentioned that the primary difficulty with FAX is the removal of the thin layer of residual droplets formed at the macula. They suggest that this layer cannot be removed using a vitrectomy probe or a flute needle. However, we don’t see any reason for this difficulty if the backflush cannula is inserted at the appropriate level of the SO infusion fluid interface and, in fact, have found good results using FAX.

One other advantage of FAX which we have not experienced in our study is that FAX cycles can allow an occult break to collect subretinal fluid, and this will reveal a subtle detachment that otherwise may have been recognized only postoperatively.

In both of our groups, SOI is positively correlated with the duration of tamponade. While some studies have shown that a prolonged SO tamponade does not lead to ocular complications, the most common recommendation is to remove SO within three-six months. However, it is important to individually evaluate every patient before removing SO tamponade to ensure that the retina has properly attached.^15^,^16^

We did not find statistically significant correlation of SOI with AL in FAX group. On the other hand, in the OFX group, we found a statistically significant positive correlation of SOI with AL (Pearson’s r = 0.394). Shihara *et al*, who use OFX, have also reported a positive correlation of residual SOI with AL.^17^ These results seemingly suggest that FAX should be considered the preferred method in cases of eyes with a longer AL.

In the OFX group, two patients (6.45%) were excluded due to re-detachment. One of these patients had the SO tamponade for three months while the other for three years. FAX group did not resulted in any re-detachment whereas there was a small (6.45%) rate of re-detachment in the OFX group. Re-detachment in cases of ROSO for various methods has been reported in the literature between 6% to 34%.^18^ In the case of a FAX, Akkan *et al* reported a re-detachment rate of 5.5%.^19^ It is to be noted that 360° laser photocoagulation was applied in all cases either preoperatively or per-operatively during ROSO. In the FAX group, one patient was exluded from consideration who had developed endophthalmitis.

This study did not find any correlation of SOI with the indication of SO tamponade, vision, diabetes, hypertension, or lens status. Some studies have shown that IOP returns to the normal range after ROSO. We, however, do see a persistent raised IOP (≥22 mm Hg) after ROSO in both groups. In the FAX group, this persistent raised IOP was seen in only 16.67% of individuals whereas in the OFX group the same was seen in 54.8% of individuals. This raised IOP may be caused by trabecular meshwork edema due to post-operative inflammation. Another reason may be the mechanical impact of infusion fluid during ROSO may split the SO droplets into much smaller drops, which are more likely to obstruct the trabecular meshwork.^20^

This study did not report any cases of post-op transient hypotony which is reported in the literature between 5% to 40% of the cases. This is likely because sutures were applied in all of our patients to close sclerostomies.^21^

Our study is the first in Pakistan to quantitatively measure the residual SO droplets while earlier studies gauged efficacy of SO removal techniques indirectly by looking at side effects resulting from residual SO. We have demonstrated that FAX is superior to OFX and would recommend it as the protocol of choice.

### Limitation

The primary limitation of our study is a relatively small sample size.

## CONCLUSION

*Fluid-air exchange group was found to be superior in reducing residual SO droplets compared with the oil-fluid exchange group. Fluid-air exchange is the preferred method as* it decreases residual SO droplets thereby decreasing SO related complications, resulting in less number of patients with a reported increased IOP and no case presenting with re-detachment.

### Author’s Contributions:

**AR:** Conception and design of study, Data collection.

**RMJ:** Manuscript writing.

**SL:** Study design, Data collection.

**SS:** Data analysis, Editing of manuscript.

**AAK:** Revision of manuscript, responsible for integrity of study.
